# Sleep

**Published:** 1856-04

**Authors:** 


### SLEEP.

Observation and scientific experiment constantly confirm the fact, that *the brain is nourished, repaired, during sleep.* If then we have not sleep enough, the brain is not nourished, and like everything else, when deprived of sufficient nourishment, withers and wastes away, until the power of sleep is lost and the whole man dwindles to skin and bone, or dies a maniac!

The practical inferences which we wish to impress upon the reader are two:

1st. By all means, sleep enough, give all who are under you sleep enough, by requiring them to go to bed at some regular hour, and to get up the moment of spontaneous waking in the morning. Never waken up any one, especially children, from a sound sleep, unless there is urgent necessity; it is cruel to do so; to prove this, we have only to notice how fretful and unhappy a child is, when waked up before the nap is out.

2. If the brain is nourished during sleep, it must have most vigor in the morning, hence the morning is the best time for study; for then, the brain has most strength, most activity, and must work more clearly. It is "the midnight lamp" which floods the world with sickly sentimentalities, with false morals, with rickety theology, and with all those harum scarum dreams of human elevation, which abnegate Bible teachings.

